# Update of Potential Biomarkers in Risk Prediction and Monitoring of Atherosclerosis in Systemic Lupus Erythematosus to Prevent Cardiovascular Disease

**DOI:** 10.3390/biomedicines11102814

**Published:** 2023-10-17

**Authors:** Dominika Blachut, Brygida Przywara-Chowaniec, Andrzej Tomasik, Tomasz Kukulski, Beata Morawiec

**Affiliations:** 2nd Department of Cardiology, Medical University of Silesia in Katowice, 41-800 Zabrze, Poland

**Keywords:** systemic lupus erythematosus, biomarkers, atherosclerosis, interferon signaling, cardiovascular risk, immune cells

## Abstract

Systemic lupus erythematosus is a chronic connective tissue disease associated with an increased risk of premature atherosclerosis. It is estimated that approximately 10% of SLE patients develop significant atherosclerosis each year, which is responsible for premature cardiovascular disease that is largely asymptomatic. This review summarizes the most recent reports from the past few years on biomarkers of atherosclerosis in SLE, mainly focusing on immune markers. Persistent chronic inflammation of the vascular wall is an important cause of cardiovascular disease (CVD) events related to endothelial dysfunction, cell proliferation, impaired production and function of nitric oxide and microangiopathic changes. Studies on pathogenic immune mediators involved in atherosclerosis will be crucial research avenues for preventing CVD.

## 1. Introduction

Systemic lupus erythematosus (SLE) is a chronic connective tissue disease associated with an increased risk of premature atherosclerosis, a 6-fold higher cardiovascular risk and even a 10-fold higher prevalence of myocardial infarction compared to the general population. It is estimated that approximately 10% of SLE patients develop significant atherosclerosis each year, which is responsible for premature cardiovascular disease (CVD) that is largely asymptomatic [1,2,3,4]. Patients with SLE are at risk for premature atherosclerosis as early as in childhood and adolescence [5,6]. CVD-related complications are the most common cause of death, with pericarditis occurring in 20–50% of patients. The first decade after the diagnosis is associated with high progression, increased disease activity and the further development of CVD complications. Classical CVD risk factors, immune background and immunotherapy are responsible for the mechanisms of progressive atherosclerosis in SLE. Subclinical atherosclerosis is characterized by endothelial dysfunction, oxidative stress-related abnormalities, increased arterial stiffness, increased apoptosis, protease expression, chronic vasculitis and dysregulation of cytokines and adipokines [7,8,9,10,11]. Persistent chronic inflammation of the vascular wall is an important cause of CVD events related to endothelial dysfunction, cell proliferation, impaired production and function of nitric oxide (NO) and microangiopathic changes. An additional important factor is connected with a significantly reduced protective role of statins in SLE compared to the general population [4,12,13,14,15]. 

To date, the following have been considered biomarkers of atherosclerosis: anti-cardiolipin antibodies, antibodies to b2-glycoprotein I, dimethylarginine, resistin, leptin, neopterin, tumor necrosis factor α (TNF-α), interleukin-1 alpha (IL-1α), vascular endothelial growth factor (VEGF), myeloperoxidase (MPO), matrix metalloproteinase-9 (MMP-9), serum amyloid A (SAA), vascular cell adhesion molecule-1 (VCAM-1), intercellular adhesion molecule-1 (ICAM-1) and E-selectin. Despite many examined factors, none of them has gained clinical use [16,17,18,19]. CVD prophylaxis for modifiable risk factors, such as hypercholesterolemia and hypertension, did not show a significant decrease in the development of CVD in SLE [20,21,22]. 

This review summarizes the most recent reports from the past few years on biomarkers of atherosclerosis in SLE, mainly focusing on immune markers. The paper also discusses the immune pathogenesis of atherogenesis. References for this paper were collected from PubMed, Clinical Key, EMBASE Platform and Web of Science. We summarized the most important reports published in the last five years (2018–2023). In the case of an insufficient number of papers from this period, the search was extended to 10 years (2013–2023).

## 2. The Pathogenesis of Atherosclerosis in SLE

The pathogenesis of atherosclerosis and the development of cardiovascular disease in SLE is associated with the activation and progression of apoptosis, expression of extracellular matrix proteases responsible for endothelial cell (EC) degradation and the prothrombotic effect of autoantibodies. This cascade of processes leads to the formation of atherosclerotic plaque. Chronic inflammation is crucial for the progression of atherosclerosis in SLE. Anti-endothelial cell antibodies and antiphospholipid (APL) antibodies may be related to endothelial damage and prothrombotic effects in SLE. Impaired protective mechanisms, such as EC repair and decreased production of protective antibodies, lead to the progression of atherogenic processes [15,23,24].

Cytokines are an important modulator of the activation of programmed cell death. They are also involved in atherosclerotic plaque formation and, indirectly, in its growth via leukocyte adhesion. Type I interferon (IFN-I), mainly IFN-α and IFNβ, is connected with atherosclerotic plaque formation in SLE via affecting neutrophil function, changing cellular metabolism, inducing apoptosis of endothelial progenitor cells (EPCs) and circulating angiogenic cells (CACs) and affecting macrophages in the atherosclerotic plaque by enhancing foam cell formation. This process leads to endothelial dysfunction and the impairment of dendritic cells (DCs) and T and B lymphocytes. By affecting the interleukin 1 (IL-1) pathway, IFN-α affects vascular repair dysfunction. IFN-α inhibits IL-1α, IL-1β, interleukin 1 receptor, type I (IL1R1) and VEGF and up-regulates the IL-1 receptor antagonist (IL-1RA). IL-1β has a protective role, while IL-18 and IL-10 negatively affect EPCs and CACs, whose significant dysfunction is found in SLE [25,26,27,28,29,30,31]. Activated ECs secrete monocyte chemotactic protein-1 (MCP-1), which attracts monocytes to the subendothelial space in which they differentiate into foam cells and phagocytize oxidized forms of low-density lipoprotein (ox-LDL). Such foam cells can actively express protein genes of proinflammatory factors, including TNF-α and interleukin-6 (IL-6). Leukocyte recruitment is also stimulated by L-homocysteine, which is induced by the expression of MCP-1 and interleukin-8 (IL-8). Endothelial damage also results from the deposition of ox-LDL, which promotes the inflammatory response by activating ECs and secreting adhesion molecules and chemokines, thus increasing endothelial permeability for monocytes. The resulting foam cells from macrophages in the endothelial layer trigger the proliferation of smooth muscle cells and lead to atherosclerotic plaque proliferation. At the same time, the reaction between platelets and ECs occurs, which results in the stimulation of IL-8 and ICAM-1 production with endothelial dysfunction and thrombosis [13,23,32,33,34]. Higher plasma VEGF levels are associated with disease progression and increased activity in SLE. VEGF correlates with the common carotid intima-media thickness (cIMT) [23,35,36,37,38]. Fibroblast growth factor 21 (FGF21), which is a protein secreted by the liver, plays a role in glucose and lipid metabolism. Studies have shown changes in FGF21 levels in SLE, which was crucial for delaying the progression of atherosclerosis. 

**Figure 1:**
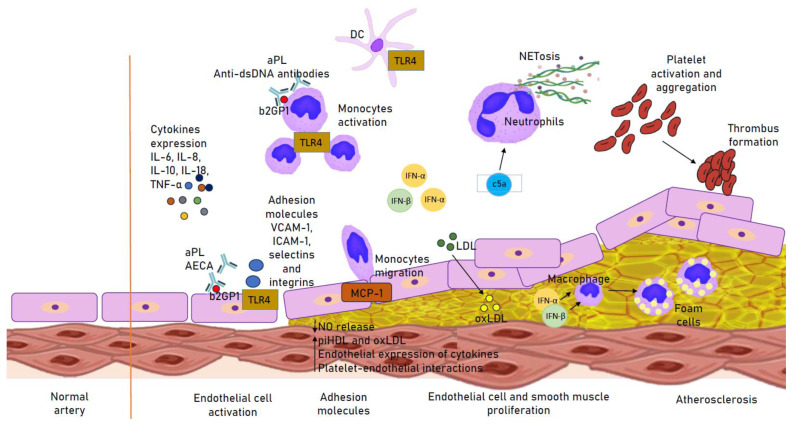
Immune mechanisms involved in the atherogenesis in SLE. Up arrow: increased secretion; Down arrow: decreased secretion. aPL, antiphospholipid antibody; b2GP1, Beta 2 Glycoprotein 1; DC, dendritic cell; IFN(α, β), interferon (α, β); (ox)LDL, (oxidized) low-density lipoprotein; MCP-1, monocyte chemotactic protein-1; piHDL, proinflammatory high-density lipoprotein; TLR4, Toll-like receptor 4.

Neutrophil extracellular traps (NETs) probably play a proatherogenic role in SLE, which is related to prothrombotic activity and the action impairing the protective effects of high-density lipoprotein (HDL) [39]. Activation of apoptotic receptors by their respective ligands (such as FasL, TNF-a and TRAIL) is essential for the deletion of activated T lymphocytes and cell death. FasL/Fas signaling plays an important role in the activation of apoptosis. Changes in the receptors are associated with impaired lymphocyte function and increased exposure to autoantigens, and the pathogenesis is believed to be due to autoimmunity. In addition, disease activity in SLE is significantly associated with increased Fas expression on the surface of T and B cells. SLE patients with CVD may have higher levels of soluble death receptors [1,40]. A summary of the pathogenesis of atherosclerosis in SLE is provided in Figure 1.

The levels of antibodies are different in SLE. They are also important in proatherogenic processes. APLs were associated with Beta 2 Glycoprotein 1 (b2GP1) expression in monocytes, showing a proliferative effect that significantly affected cIMT [41,42,43]. Anti-b2GP1 is involved in releasing IL-1β and Th17/Th1 and activating EC via the Toll-like receptor 4 (TLR4). It also increases the expression of adhesion molecules (such as VCAM-1, ICAM-1, selectins and integrins). IgG anti-b2GP1 antibodies recognize anti-b2GP1 complexes with ox-LDL, which facilitates macrophage passage and foam cell formation. Anti-b2GP1 affects T helper (Th) cells that secrete IL-17 and IFN-α in response to stimulation and formation of local inflammation by the presence of TH17/Th1 in the atherosclerotic plaque and its instability with the subsequent rupture. The presence of IL-23 may also stimulate Th17. APL is most likely involved in the induction of thrombosis by inhibiting the activity of von Willebrand factor [44,45,46,47]. Anti-dsDNA antibodies are associated with the abnormal activation of cells involved in non-specific immunity (mainly monocytes and neutrophils). They induce processes related to NETs, i.e., NETosis in neutrophils and apoptosis in monocytes, and they also affect inflammation and activate ECs. NETosis is involved in the production of enzymes that cause HDL oxidation by modifying the molecule to a proatherogenic lipoprotein. Anti-dsDNA antibodies are significantly associated with endothelial dysfunction, proatherogenic dyslipidemia and acceleration of atherosclerosis [48,49,50]. Anti-endothelial cell antibodies (AECAs) are a group of antibodies against E-proteins. Their action is associated with the induction of the release of proinflammatory factors and adhesion molecules (E-selectin, ICAM-1, VCAM-1, cytokines [IL-1, IL-6, IL-8] and chemokines [MCP-1]) through the activation of NF-kB and vasculitis [51].

## 3. Biomarkers

### 3.1. Immune Cells

Immune cells represent an important line of research in the search for a suitable molecule as a potential biomarker of early atherosclerosis in SLE. Of the cascade of molecules involved in atherosclerotic plaque formation and influencing the development of inflammation, only a few of them can be considered potential biomarkers. Activation of NF-kB as a transcriptional regulator results in increased production of interleukin-6 (IL-6) and ICAM-1 [52]. Studies on IL-6 as a biomarker of early atherosclerosis showed inconclusive results and its significant value was not confirmed [53,54]. 

Interleukin-18 (IL-18) shows atherosclerosis-promoting effects in ECs. It is increased by IFN dysregulation. IL-18 levels are elevated in SLE and significantly correlate with EPCs and atherosclerotic plaque thickness [55,56,57,58]. T lymphocytes are responsible for producing proinflammatory cytokines, such as IFN-γ and IL-17, and may contribute to atherosclerotic plaque formation. Additionally, regulatory T cells (Tregs) have direct atherogenic effects [59,60,61,62]. These findings were confirmed in mouse and human models [63,64]. Overexpression of Pbx1d in CD4+ T cells was related to the thickening of the arterial wall. However, this was not confirmed in other clinical studies [65]. Some studies found that interleukin-2 (IL-2) levels were lower in SLE. IL-2 therapy reduced CD4+ T-cell activity and active inflammation [66,67]. VCAM-1 may also act as a potential biomarker [68,69,70]. In their study on CXCR3+ T-follicular helper (TFH) cells isolated from Apoe mice, Ryu et al. found higher expression of genes related to inflammatory responses and induction of IgG2c in SLE. The atherogenic environment induced IL-27 from dendritic cells in a Toll-like receptor 4 (TLR4)-dependent manner. Blockade of IL-27 signaling reduced TFH cell responses in atherogenic mice. In turn, atherogenic dyslipidemia augmented TFH cell responses and subsequent IgG2c production in a TLR4 and IL-27-dependent manner [71]. Despite many studies, interleukins do not currently have clinical applications as biomarkers due to the complex nature of their interactions.

IFN is one of the most important cytokines in SLE. Type 1 IFN is secreted mainly by dendritic cells and low-density granulocytes. It is associated with EC dysfunction in SLE. IFN expression increases the activation of Toll-like receptors 7 and 9. It causes destabilization of the atherosclerotic plaque. Activation of IFN pathways was associated with the progression of atherosclerosis in SLE [25,68,72,73,74,75]. However, study findings are inconclusive, and there is no clear answer as to whether IFN could be used as a biomarker of atherosclerosis [76,77,78]. Soluble tumor necrosis factor-like weak inducer of apoptosis (sTWEAK) increases IFN expression in mononuclear cells and may be a potential biomarker for SLE with nephritis and CVD [15,79,80]. Levels of sTWEAK are also significantly correlated with the presence of plaque in coronary arteries [81]. 

In a study on apolipoprotein E-deficient mice, the use of anti-TWEAK antibodies resulted in significant inhibition of atherosclerotic plaque formation [82]. Although some anti-TWEAK therapies are tested in animal clinical trials, none of them has found a potential use in the prevention of atherosclerosis. Therefore, further studies on humans are warranted.

NETs are chromatin fibers released from dying neutrophils by type I IFN stimulation. Increased NETs are associated with atherosclerosis progression and EC apoptosis via MMP2 stimulation, platelet activation and serine protease release. NETs are indirectly involved in the production of IFN, IL-1 and IL-18 by low-density granulocytes and dendritic cells. They also enhance NETosis [37,83,84,85]. Hakkim et al. reported that impaired NET degradation occurred in SLE, which is associated with the presence of antibodies against NETs and DNase 1 endonuclease [86]. Theoretically, determining NETs may be beneficial. However, interactions with other pathways should be considered. In addition, patients with SLE showed higher levels of low-density granulocytes (LDGs). This subset of neutrophils was linked to higher levels of NETs [82].

Vascular adhesion protein-1 (VAP-1) is a proinflammatory protein in the cell membrane that facilitates leukocyte migration after TNF-α, IFN-γ or IL-1β stimulation [87]. VAP-1 showed higher values in SLE and could be a potential marker of atherosclerosis [88]. The positive correlation between VAP-1 and atherosclerosis was confirmed in humans [89,90] and animal models [91,92]. 

Metabolites of the arachidonic acid pathway, such as leukotriene B4 (LTB4), the oxidized lipids 9/13-hydroxyoctodecadienoic acid (HODE) and 5-hydroxyeicosatetraenoic acid (5-HETE), may be potential markers of atherosclerosis. LTB4 is associated with leukocyte adhesion to ECs and the release of free radicals [93,94,95]. 9-HODE and 13-HODE are also involved in atherogenesis [93,96]. 

B2 cells may have a significant impact on the mechanism of atherosclerosis. B2 cells exhibit atherogenic effects, while B1 cells are seen as protective. Selective inhibition of B2 with anti-B therapy appears to be a promising therapeutic strategy against the development of atherosclerosis in SLE. The cytokine B-cell activating factor (BAFF) is responsible for B2 B-cell maturation. These cells play an antiatherogenic role in SLE. Inhibition of the BAFF-BAFF receptor (BAFFR) on B cells and EPCs results in atherogenic effects. When the BAFF was inhibited in macrophages, foam cell formation was reported and a proatherogenic effect was found with coexisting lipid disorders [97,98,99,100,101,102]. 

BAFF is associated with subclinical atherosclerosis, and a significant correlation with higher cIMT values was observed. Biologic drugs (anti-BAFF), such as belimumab, significantly reduced the risk of CVD [102,103,104]. BAFF and a proliferation-inducing ligand (APRIL) are involved in regulating B-lymphocyte activation, and they both play a role in atherogenesis [105]. BAFF and APRIL levels were positively correlated with the increase in cIMT and arterial wall thickening. According to Shater et al., APRIL could be a sensitive potential biomarker for detecting early atherosclerosis [106,107]. Salazar-Camarena et al. suggested a potential role of APRIL as a marker of atherosclerosis [105].

TRAIL-receptor-2 (TRAIL-R2) is significantly associated with CVD [108]. Studies confirmed the association between higher TRAIL-R2 levels and glucocorticosteroid treatment, atherosclerotic calcified plaque in the carotid artery, and twice-as-high values in SLE, which resulted in a higher risk of CVD in these patients. In SLE patients, TRAIL-R2 receptor levels were lower in patients without CVD than in those with CVD [1,109]. 

Endothelial progenitor cells (EPCs) play a role in neovascularization and are responsible for normal endothelial function. EPC levels are significantly decreased in SLE. EPCs may be a potential biomarker in patients at risk of developing CVD. Castejon et al. showed that lower EPC levels are correlated with increased arterial stiffness and significantly increased the risk of atherogenesis and CVD. Studies showed the reversibility of the decrease in EPC levels after treatment with type 1 IFN and BAFF [110,111,112,113].

TNF-α plays a role in promoting the expression of adhesion molecules. Higher TNF levels were found in SLE. In their immunohistochemical examination, Pomara et al. revealed a positive reaction in cardiac myocytes for antibodies anti-TNF-α and interleukins (IL-8, IL-10 and IL-15) associated with subclinical atherosclerosis and myocyte disorders. TNF-α was positively correlated with CVD risk factors in adolescent and adult populations [114,115,116,117].

The release of proinflammatory factors via the Toll-like receptor (TLR) signaling pathway due to overactivation of the receptor or dysregulation of endogenous inhibition of TLR signaling can lead to chronic inflammation, autoimmunity and myocardial dysfunction. TLR2 is associated with the activation of macrophages and DCs by nucleosomes related to high mobility group protein 1 (HMGB1). It may be involved in the formation of autoantibodies through the activation of antigen-presenting cells (APCs) [118]. 

TLR2 signaling is associated with left ventricular dysfunction and activation of the Toll interleukin 1 receptor (TIR) domain-containing adaptor protein (TIRAP) in myocardial ischemia and subsequent reperfusion. TLR2 and TLR4 expression was found in atherosclerotic coronary artery plaque [119,120,121]. The increase in TLR2 expression in CD14+ monocytes may be influenced by hypoxia [52]. Fibroblast growth factor 21 (FGF21) may be a promising biomarker, and its decrease shows lower levels of progenitor cells in SLE [104,122]. Its potential role was observed in mouse models [123,124]. However, further studies on humans are warranted. 

Specific soluble mediators, such as annexin A5, platelet and endothelial cell adhesion molecule 1 (PECAM1/CD31), CD163+ macrophages and activated leukocyte cell adhesion molecule (ALCAM/CD166), are elevated in SLE. These mediators are significantly involved in EC dysfunction. Valer et al. demonstrated that annexin A5 levels correlated with EC dysfunction and IMT. CD163+ macrophages are involved in atherosclerotic plaque progression via the CD163/HIF1α/VEGF-A pathway [125,126,127,128]. However, there are no extensive studies proving the use of annexin A5, PECAM1/CD31 or CD163+ macrophages.

β2-microglobulin (β2MG) is a plasma protein associated with atherosclerotic plaque formation. Leffers et al. analyzed atherosclerotic plaques and coronary artery calcification in relation to plasma β2MG levels. Calcified plaque (CP) and coronary artery calcification were observed in 20% and 39% of patients, respectively. CP or coronary artery calcification was associated with the highest quartile of plasma β2MG, which may be a potential biomarker of atherosclerosis in SLE [129]. However, studies confirmed that serum β2-MG levels were higher in SLE and were correlated with SLE disease activity. Therefore, serum β2-MG levels may not be a biomarker of atherosclerosis in SLE [130].

Elevated levels of complement products are associated with increased disease activity, and they play a role in endothelial damage together with immune complexes. The C3 component of complement is a biomarker routinely used in monitoring disease activity. In addition, C3 is associated with subclinical atherosclerosis, mainly in small arteries. In animal models, C3 was deposited in the endothelium, and by binding to elastin and collagen fibers, it led to increased stiffness of blood vessels. A role in the pathogenesis of CVD was associated with C4 deficiency. The levels of cell-bound complement activation products (CB-CAPs), namely B cell C4d (BC4d) and erythrocyte C4d (EC4d), are specific to SLE as diagnostic, monitoring and prognostic biomarkers of disease activity. In addition, the formation of terminal complement complexes as a biomarker indicates the cascade activation and the formation of vasculitis. C5a activates neutrophils with subsequent activation of the extrinsic coagulation cascade [131]. Molecule pentraxin 3 (PTX3), which is associated with complement activation in the alternative pathway and vasculitis, may also be a biomarker [114,132,133,134]. PTX3, which is a marker of atherosclerosis or local vasculitis in SLE, is secreted by dendritic cells and ECs in response to inflammation. It is associated with VCAM-1 and vWf [79,135,136].

It should be noted that PTX3 levels are also elevated in immune-mediated inflammation and correlated with disease activity or glucocorticosteroid therapy. High PTX3 levels may be a potential risk factor for vascular involvement in SLE patients. However, PTX3 is not specific to proatherogenic activation only [137]. A summary of potential biomarkers of atherosclerosis is provided in Table 1.

### 3.2. Inflammatory Enzymes

MMP-9 is involved in the induction of endothelial cell apoptosis. Endothelial dysfunction is correlated with MMP-2 activation by MMP-9 in NETs. Complexes with MMP-9 and anti-MMP-9 were found in SLE. Anti-MMP-9 antibodies induced NETosis and increased MMP-9 activity. MMP-9 antibodies may be a marker of atherogenesis [138]. However, one study did not confirm this thesis because MMP-9 was not significantly associated with coronary atherosclerosis after considering the Framingham risk score [16].

### 3.3. Antibodies

APL antibodies are divided into antiphosphatidylethanolamine (aPE) and antiphosphatidylserine (aPS) antibodies. The most common aPS antibodies include lupus anticoagulant (LAC), anticardiolipin (aCL) and anti-β2-glycoprotein-1 (anti-β2GPI) antibodies. They show affinity for anionic membrane phospholipids and plasma proteins by binding phospholipids. The presence of APL may be associated with thrombus formation, which can lead to thrombosis and thrombocytopenia. These antibodies are related to early atherosclerosis and correlate significantly with cIMT in clinical studies [40,139,140,141]. A summary of impact of antibodies on endothelial dysfunction and atherogenesis in SLE is provided in Table 2.

### 3.4. Other Biomarkers

High-sensitivity troponin (hs-Trop) is an essential marker in acute coronary syndrome. Patients with SLE have a high prevalence of subclinical myocardial injury, which is associated with the release of hs-Trop. Concentrations of hs-Trop in SLE are below the threshold for acute coronary syndrome (>30 ng/L). Therefore, there are no significant associations between hs-Trop and markers of cardiac structure. Troponin is released in patients with active inflammation, which can lead to cell necrosis. Higher levels of hs-Trop may be related to the presence of atherosclerotic plaque in SLE patients with an apparently low risk of CVD [142,143,144].

The N-terminal fragment of pro-B-type natriuretic peptide (NT-proBNP) is important in the diagnosis and risk stratification of congestive heart failure. ProBNP is released from cardiac myocytes in response to volume and pressure overload. It includes NT-proBNP and BNP, which is involved in increasing diuresis and natriuresis, while NT-proBNP does not affect changes in the body. NT-proBNP and BNP in SLE may correlate with organ dysfunction and disease duration. Goldberg et al. showed that NT-proBNP was not associated with atherosclerosis or arterial stiffness but could be a marker of ventricular dysfunction [145].

NO is released from endothelial cells by NO synthase inhibited by asymmetric dimethylarginine (ADMA) and has vasodilatory effects. Decreased availability of NO is associated with endothelial dysfunction and an early stage of the atherosclerotic process. It also inhibits platelet and leukocyte aggregation to the endothelium and regulates vessel blood flow. Higher levels of ADMA are reported in chronic heart failure and hypertension. In a study by Kiani et al., levels in the highest quartile were associated with a nearly 4-fold increase in CVD risk [18]. In the CARDIAC study, ADMA levels > 1.75 μM/L were associated with an almost 7-fold increase in CVD risk and coronary artery calcification, thus a higher risk of coronary artery disease. [138]. In animal models, the absence of ADMA led to the rapid development of atherosclerosis. L-arginine supplementation may reverse the atherosclerotic process [18].

Dysfunctional proinflammatory high-density lipoprotein (piHDL) is the oxidized form of HDL. Both piHDL and ox-LDL play an essential role in atherosclerotic plaque formation. HDL, which is usually considered antiatherogenic, plays the opposite role in this case. The presence of piHDL is significantly associated with atherosclerotic plaque formation and the increase in intima-media thickness (IMT). Studies showed that piHDL was involved in monocyte chemotaxis and secretion of MCP-1 and TNF [34,146,147,148].

Endocan is a marker of angiogenesis and endothelial cell activation associated with leukocyte adhesion and migration through ECs. Higher concentrations of endocan are found in SLE. Studies found that it was significantly associated with cIMT. It may be a potential biomarker of early atherosclerosis [34,149,150].

Fetuin-A is a glycoprotein whose decreased levels may be involved in CVD progression [151]. This glycoprotein is a vascular factor involved in the deposition of hydroxyapatite in arterial walls and prevents the precipitation of calcium and phosphorus in serum. Fetuin-A levels are inversely associated with the presence of atherosclerotic plaque and the progression of atherosclerosis [152]. Studies found that fetuin-A levels were decreased in SLE and inversely correlated with cIMT. Ross et al., Mosa et al. and Atta et al. confirmed that fetuin-A could be a potential biomarker of atherosclerosis in patients with SLE [151,152,153].

## 4. Conclusions

The high cardiovascular risk of SLE remains a significant concern. SLE requires the monitoring of inflammatory activity in addition to classical cardiovascular risk factors to prevent atherogenesis. Studies on pathogenic immune mediators involved in atherosclerosis will be crucial research avenues for preventing CVD. Maintaining normal endothelial function is central to the antiatherogenic mechanism. Potential immune biomarkers may provide an appropriate direction for predicting atherogenesis and CVD risk in patients with SLE. It should, however, be remembered that complex immune pathways pose a significant clinical problem when a single marker is isolated. The optimal pharmacological combination can offer encouraging effects in preventing atherosclerosis, mainly in terms of combining immunosuppressive therapy with anti-inflammatory drugs. Targeted therapy for inflammation may hold promise in the prevention and treatment of CVD. However, there is still a lack of data on the effectiveness of therapy in preventing atherosclerosis in SLE. Therefore, further studies are warranted.

## Figures and Tables

**Table 1:** A summary of potential biomarkers of atherosclerosis.

Name of the Biomarkers	Significant Value in Atherosclerosis	Comment
IL-2	insufficient data	reduced active inflammation
Il-6	not confirmed	inconclusive results
IL-18	possible	increased by IFN dysregulation
T lymphocytes/Tregs	possible/confirmed	producing proinflammatory cytokines (IFN-γ, IL-17)
Pbx1d	not confirmed	
VCAM-1	possible	
TFH	possible	study on mice
IFN	possible	Activation of IFN pathways was associated with the progression of atherosclerosis
sTWEAK	possible	increases IFN expression
NET	possible	released from dying neutrophils by type I IFN stimulation; indirectly involved in the production of IFN, IL-1, IL-18
LDG	possible	linked to higher levels of NET
VAP-1	confirmed	confirmed in humans and animal models
LTB4, HODE, 5-HETE	possible	further research required
BAFF	possible	correlation with higher cIMT
APRIL	possible	increase in cIMT and arterial wall thickening
TRAIL-R2	Possible	Association with glucocorticosteroid treatment and atherosclerotic calcified plaque in the carotid artery
EPC	possible	correlated with increased arterial stiffness
TNF-α	possible	positively correlated with CVD risk factors
TLR2/TLR4	possible	expression was found in atherosclerotic coronary artery plaque; influenced by hypoxia
FGF21	possible	potential role was observed in mouse models; further studies on humans are warranted
Annexin A5	possible	correlated with EC dysfunction and IMT
β2MG	insufficient data	correlated with SLE disease activity
C3	possible	associated with subclinical atherosclerosis
PTX3	confirmed	elevated in immune-mediated inflammation and correlated with disease activity or glucocorticosteroid therapy

IL-2, Interleukin-2; IL-6, Interleukin-6; IL-18, Interleukin-18; Tregs, regulatory T cells; VCAM-1, vascular cell adhesion molecule 1; TFH, T-follicular helper; IFN, Interferon; sTWEAK, soluble tumor necrosis factor-like weak inducer of apoptosis; LDG, low-density granulocytes; VAP-1, Vascular adhesion protein-1; LTB4, leukotriene B4; HODE, oxidized lipids 9/13-hydroxyoctodecadienoic acid; 5-HETE, 5-hydroxyeicosatetraenoic acid; BAFF, the cytokine B-cell activating factor; APRIL, proliferation-inducing ligand; TRAIL-R2, TRAIL-receptor-2; EPCs, endothelial progenitor cells; TNF-α, tumor necrosis factor α; TLR, Toll-like receptor; FGF21, fibroblast growth factor 21; β2MG, β2-microglobulin; C3, C3 component of complement; PTX3, molecule pentraxin 3.

**Table 2:** Mechanism of impact of antibodies on endothelial dysfunction and atherogenesis in SLE.

Name of the Antibody	Impact on Endothelial Dysfunction	Atherogenic Effect
LAC	+	+
aCL	+	+
anti-β2GPI	+	+
Anti-dsDNA	+	+
AECA	+	
Anti-HDL		+
IgM anti-OxLDL		−
IgG anti-oxLDL	+	+
Anti Lp(a)		+
Anti ApoA-I		+
IgM anti-PC		−
IgM anti-MDA		−

Plus: positive impact, minus: negative impact. AECA, anti-endothelial cell antibodies; LAC, lupus anticoagulant; aCL, anticardiolipin; anti-β2GPI, anti-β2-glycoprotein-1 antibodies; Anti-dsDNA, anti-double stranded DNA; Anti-HDL, anti-high-density lipoprotein antibodies; anti-OxLDL, anti-oxidized LDL antibodies; Anti Lp(a), anti-lipoprotein(a) antibodies; Anti Lp(a), Apolipoprotein A-I antibodies; anti-PC, anti-phosphorylcholine; anti-MDA, anti-malondialdehyde.

## Data Availability

Data is contained within the article.
